# Hygrothermal monitoring of replacement infill panels for historic timber-frame buildings: initial findings

**DOI:** 10.14324/111.444/ucloe.000039

**Published:** 2022-08-26

**Authors:** Chris J. Whitman, Oriel Prizeman, Pete Walker, Iain McCaig, Soki Rhee-Duverne

**Affiliations:** 1 Welsh School of Architecture, Cardiff University, Cardiff, UK; 2 Department of Architecture & Civil Engineering, University of Bath, Bath, UK; 3 Historic England, London, UK

**Keywords:** interstitial hygrothermal behaviour, moisture content, monitoring, traditional timber frame, energy retrofit

## Abstract

Energy retrofits aim to improve the thermal performance of buildings’ external envelopes. With buildings of traditional construction there exists the risk that these improvements may lead to interstitial condensation and moisture accumulation. For historic timber-framed buildings, this potentially exposes the embedded historic timbers to conditions favouring fungal decay and insect infestation. Hygrothermal digital simulations can assess this risk, but these have limitations, especially regarding the study of historic and traditional materials, due to a lack of accurate material data. The research presented in this paper therefore uses the monitoring of physical test panels to examine the performance of four different infill solutions. These are, traditional wattle and daub, a composite of wood fibre and wood wool boards, expanded cork board, and hempcrete. The article focuses on the design and construction of the test cell and presents initial results from the first year of monitoring, following the initial drying phase. These showed no evidence of interstitial condensation in any of the panel build-ups, with increases in moisture content correlating directly with climatic measurements of wind-driven rain. Infill materials with low moisture permeability were seen to produce higher moisture contents at the interface with the external render due to the concentration of moisture at this point. Those panels finished in the more moisture permeable lime-hemp plaster, overall present lower moisture contents, with reduced drying times. The use of perimeter, non-moisture permeable, sealants would appear to potentially trap moisture at the junction between infill and historic timber-frame. The monitoring work is ongoing.

## Introduction

In order to meet the decarbonisation targets set by the UK Government to bring all greenhouse gas emissions to net zero by 2050 [1], it is necessary to address the performance of our existing building stock, including those of traditional construction generally built pre-1919. It is, however, important that improvements to the thermal performance of these buildings’ external envelopes do not lead to unintended consequences [2]. To date the majority of research in this field has focused on solid masonry construction [3,4]. However, for historic timber-framed buildings, which account for 8% of the pre-1850 housing stock [5], with over 68,000 of these buildings surviving in the UK [6], changes to the hygrothermal performance of their exposed timber-framed walls could increase the risk of fungal decay and insect attack. This article presents research, funded by Historic England, that aims to assess this risk of degradation of the historic timber members.

### Aims and objectives

The research aims to establish the risk of interstitial condensation and moisture accumulation within four potential replacement infill panels for timber-framed buildings: a) traditional wattle and daub; b) expanded cork board; c) a composite of wood fibre and wood wool boards; and d) hempcrete. Thermal performance and moisture content are being monitored over a minimum of 2 years, with hygrothermal conditions compared to those favourable for fungi and insects known to endanger hardwood frames. In the future, measured results will also be compared to those arising from digital hygrothermal simulation using WUFI^®^ Pro (Fraunhofer Institute for Building Physics, Stuttgart, Germany). The results will be used to corroborate previous research by the authors using in situ monitoring [7] and laboratory testing [8].

## Traditional timber-framed construction in the UK

**Figure 1:**
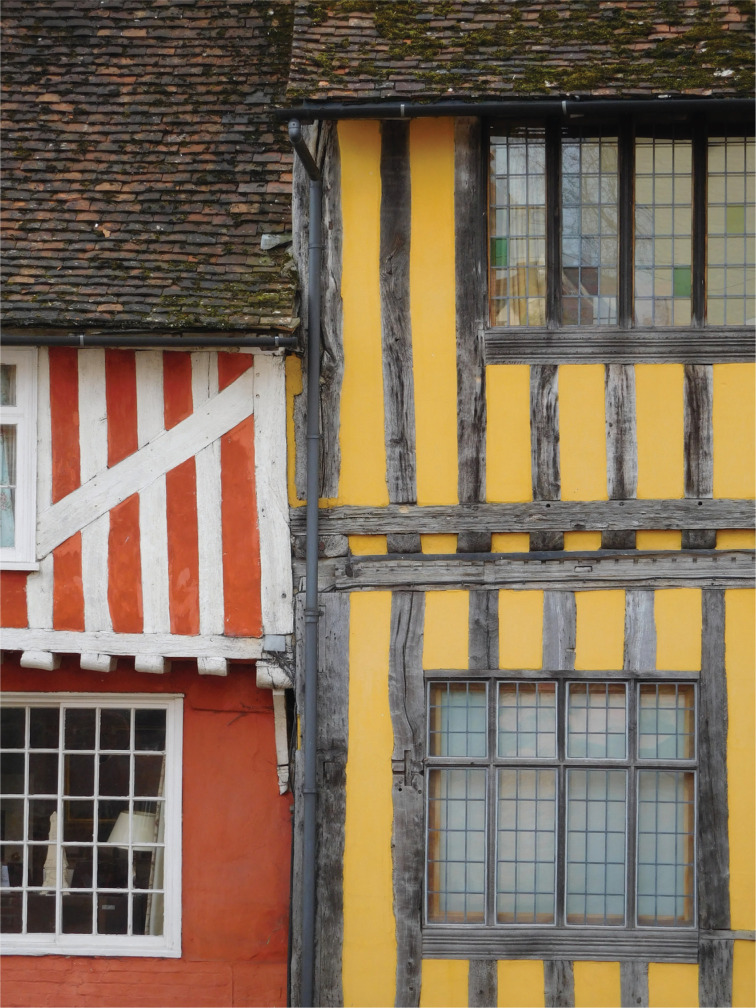
Fifteenth century timber framing, The Manor House (left) and 53 Church Street (right), Lavenham, Suffolk. Source: [6].

**Figure 2:**
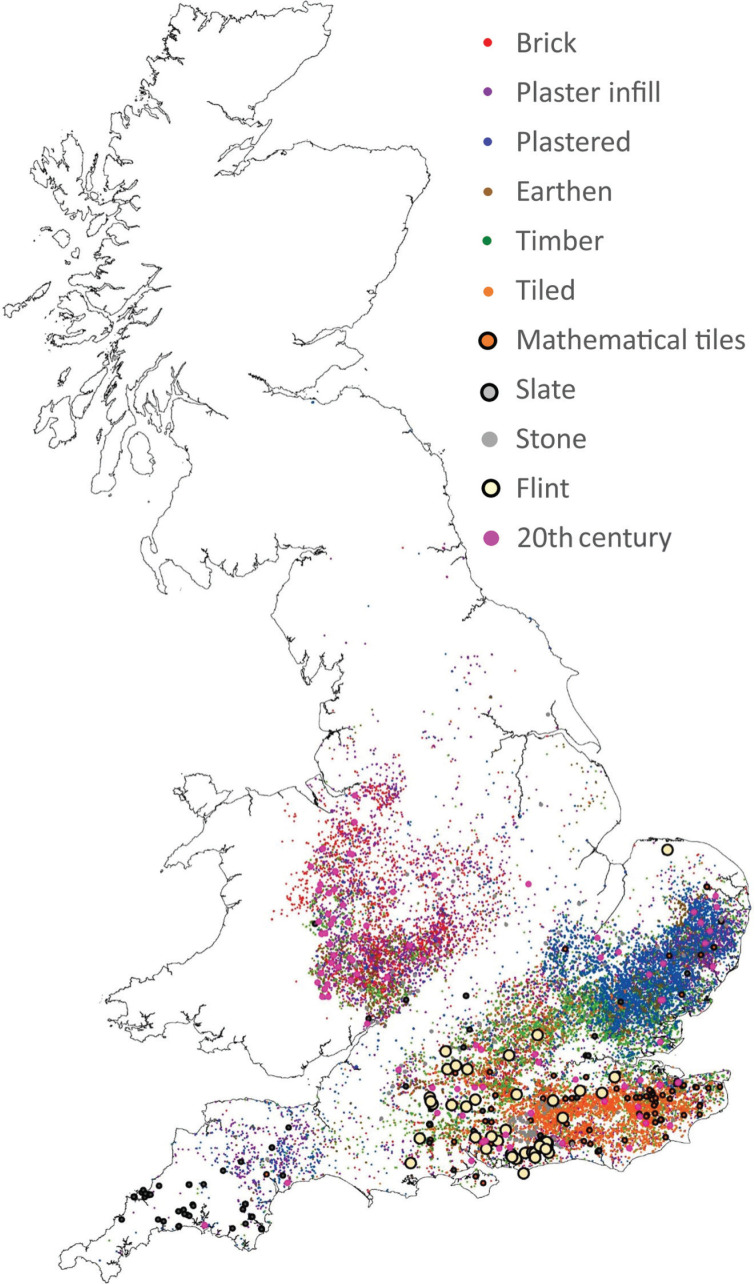
Distribution of exposed timber-framed buildings in Great Britain, classified by panel infill and cladding type. Source: Authors’ own based on [10,11].

Traditional timber-framed buildings in the UK (Fig. 1) are most commonly constructed from oak, with some examples also found in elm and other native hardwoods [9]. The timbers form a framework, which is then infilled in a variety of materials, these varying depending on the age and geographical location of the building (Fig. 2).

The timber frame is often left exposed both internally and externally, forming perhaps one of their most characteristic aesthetic heritage features, but also creating specific technical issues when considering their energy retrofit. In order to maintain the visual character of the buildings, this prohibits the use of more commonly used retrofit solutions of external wall insulation (EWI) and internal wall insulation (IWI) and restricts the introduction of insulation to the replacement of the infill panels, and only when the historic infill is beyond repair or has already been replaced with modern materials [12]. At the same time, the exposed junction between the timber frame and the infill panel is inherently a weak spot with regards to creating air and moisture movement. Historically this junction would have been sealed on a regular basis by the application of limewash across both frame and panel [13]; however, this is no longer common practice.

## The risks of energy retrofitting traditional timber-framed buildings in the UK

As with all energy retrofits, the introduction of thermal insulation will change the hygrothermal behaviour of the building envelope. This has the potential to lead to interstitial condensation both within materials and at the interface between materials, and alter the drying ability of construction elements, both of which can result in an increase in moisture content. For historic timber-framed buildings, an increase in the moisture content of the embedded timbers could create hygrothermal conditions conducive to the damaging processes of biological agents such as insects and fungi, leading to reduced structural integrity and, ultimately, loss of historic fabric. These biological agents do, however, have optimum hygrothermal conditions (Table 1). If these can be avoided, the risk is reduced.

**Table 1.** Optimum hygrothermal conditions for common UK biological timber threats [14]

Common name	Beetle and their larvae				Fungi		
	Powderpost	House longhorn	Woodworm	Deathwatch	Dry rot	Oak rot	Cellar
Latin name	*Lycus linearis Goeze* and *Lyctus brunneus*	*Hylotrupesw bajulus*	*Anobium punctatum*	*Xestobium rufovillosum*	*Serpula lacrymans*	*Coniophora puteana*	*Coniophora puteana*
Moisture content (%)	8–25	15–25	>12	>15	>26	>28	>25
Temperature (°C)	26	20-30	22	>10	17–23	5–40	20–32

### Previous work by the authors to assess these risks

Initially, digital interstitial hygrothermal simulations, using the software WUFI^®^ pro 5.3, were undertaken to investigate hygrothermal conditions created by a range of potential retrofit solutions suggested by guidance documents [15,16]. Simulations were undertaken for a range of orientations, and six distinct UK geographical locations where a significant number of surviving historic timber-framed buildings can be found. These were Suffolk, Essex, Kent, Herefordshire, Devon and a rare northern example in Cumbria. The results suggested that whilst the material properties of the replacement infill materials, orientation and climatic conditions, all had an impact on the resulting moisture content, no prolonged exposure to hygrothermal conditions favourable for biological attack, as defined in Table 1, were identified [16]. There was, however, uncertainty over the validity of the results due to the lack of material property data of some retrofit and historic materials, and the fact that these simulations represented idealised conditions with homogeneous layers well in the heterogeneous reality.

In 2017 the lead author undertook in situ monitoring at a 16th century historic timber-framed farmhouse in Suffolk UK [7]. The building had been cement rendered externally in the 1950s and had undergone a poorly considered energy retrofit in 2005, with the replacement of lath and plaster infill panels with rigid polyisocyanurate boards (PIR). Interstitial hygrothermal monitoring over a period of a year showed favourable conditions were being met for deathwatch beetle for almost 17,000 hours, accompanied by approximately 160 hours of conditions favourable for dry rot and cellar rot [7]. A smaller number of hours were also recorded of conditions favourable for other wood-boring insects.

In order to address some of the limitations of both digital simulations and in situ monitoring, three physical mock-up replacement infill panels were monitored under laboratory conditions [8]. The panels were installed to fill apertures in the dividing wall between two climatically controlled chambers at the University of Bath’s Building Research Park. The frames were constructed from reclaimed oak and the infill materials monitored were traditional wattle and daub, a composite of wood fibre and wood wool boards [14], and expanded cork board. All panels were finished on both sides in lime render. The interstitial temperature and moisture content were monitored in the centre of the panel and at the interface between the infill and a reclaimed oak frame at three depths, 10 mm, 50 mm and 90 mm, for a period of 3 weeks under steady state conditions (external chamber 5°C/80%, internal chamber 21°C/70% relative humidity [RH]). These conditions had been defined using Glaser calculations [17] as those likely to create interstitial condensation. Following these 3 weeks, a further 2 weeks were monitored using external temperatures following a diurnal cycle (5°C/94%–12°C/61%) that more closely replicated real-life conditions. The measured climatic data from the two chambers was also used in one-dimensional (1D) and two-dimensional (2D) digital interstitial hygrothermal simulations using WUFI^®^ Pro 5.3 and WUFI^®^ 2D. The in situ measurements showed that under forced steady state conditions interstitial condensation did occur in the wood fibre/wood wool composite panel, accompanied by an increase in moisture content towards the outer face of all panels (8). This was not, however, measured to occur during the 2-week period of cyclical conditions. Whilst both the 1D and 2D simulations did predict the interstitial condensation, the increase in moisture content towards the outer face of the panels was not anticipated. Significant disagreements between simulations and measured results and between 1D and 2D simulations were also encountered.

Due to technical and financial constraints, the previously described monitoring of physical mock-up panels was limited to a total of 5 weeks. In order to repeat the experiment over a longer timescale, with panels exposed to real climatic conditions, funding was sought and gained from Historic England’s Heritage Protection Commission. There follows the presentation of the design and implementation of this experiment, accompanied by analysis of the results of the first year of monitoring.

## Methodology

### Test cell design and construction

**Figure 3:**
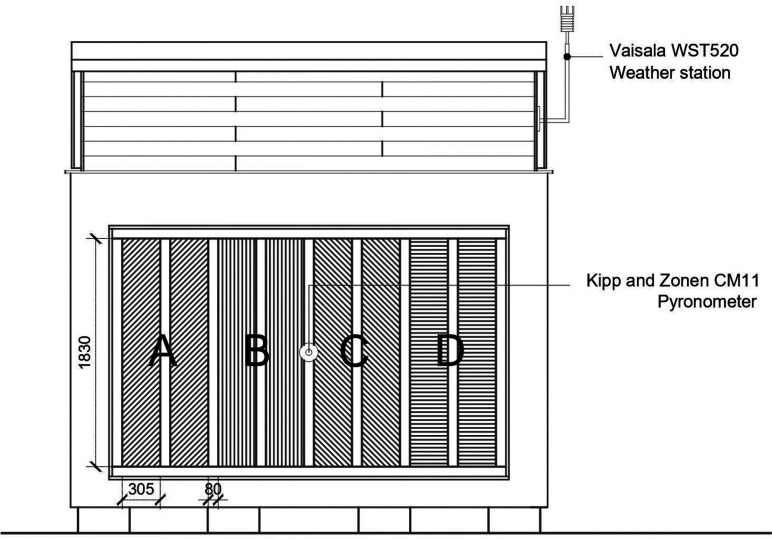
North elevation of test cell showing pairs of panels. Source: Authors’ own, 2017.

**Figure 4:**
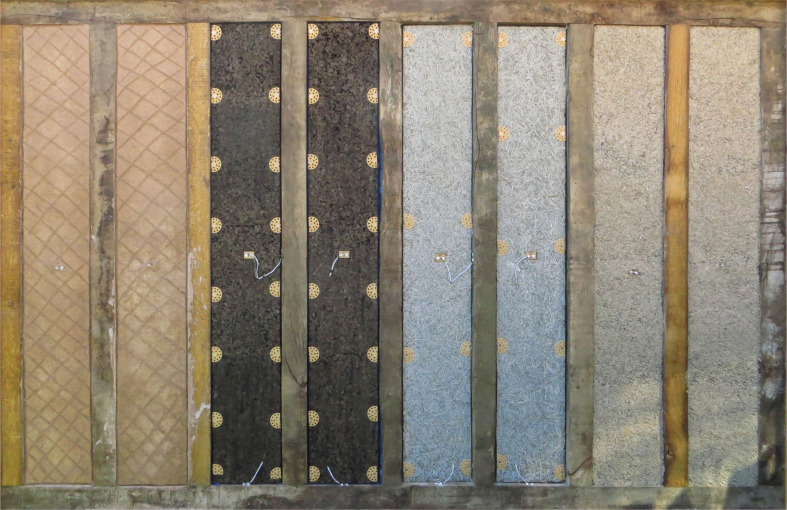
Photograph of panels prior to rendering. Source: Authors’ own 2019.

A test cell with a controlled internal environment (internal dimensions 3.5 wide × 1.9 deep × 2.2 high) was constructed at Cardiff University. Test panels form the north wall of the cell and are exposed externally to the Cardiff climate. The dimensions of the test panels were determined using the results of a study of a representative sample of 100 historic timber-framed buildings with exposed timber frames [6]. This showed 53% to be square framed (of approximately equal width and height) and 47% close studded (tall thin panels). The average size of the square panels was 785 mm × 950 mm (width × height) and the close studded 305 mm × 1830 mm (width × height), approximately 1 ft wide by 6 ft tall. Given the configuration of the test cell, it was decided to construct close studded panels, allowing the monitoring of eight adjacent panels all at the same height above ground level (Fig. 3). This array of eight panels was constructed using reclaimed oak, and allowed for the monitoring of pairs of panels of four different infill solutions (Fig. 4).

**Figure 5:**
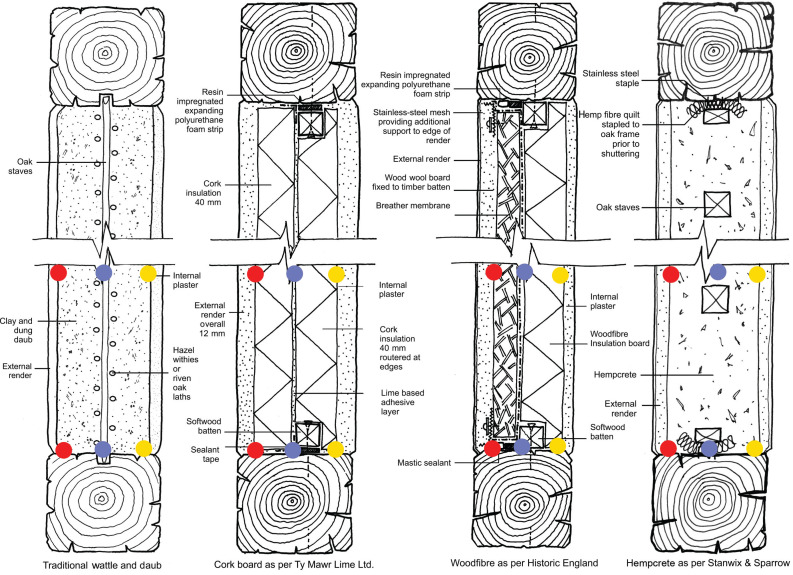
Sections showing panel infill details and monitoring locations. Red, external I, blue, central I and yellow, internal (i). Source: Author’s own based on [14,18].

The infill solutions were the three previously studied solutions: a) wattle and daub; b) expanded cork board; and c) composite wood wool and wood fibre; in addition to d) hempcrete, a construction technique originally developed in France in the 1980s specifically for the retrofit of historic timber-framed buildings [18] and recommended for such work in a number of publications [14,19]. Details of the infill solutions are shown in Fig. 5.

The first three panels were constructed in situ by Royston Davies Conservation Builders (Leominster, UK), whilst the hempcrete was installed by UK Hempcrete (Chesterfield, UK), both companies with a reputation for high-quality workmanship and conversant with working on historic buildings. The use of these professionals aimed to replicate as close as possible real-life scenarios. One of each pair of panels was finished internally and externally with a natural hydraulic lime plaster NHL 3.5 (Secil™ [Seciltek, Lisbon, Portugal]), whilst the other was finished in a non-hydraulic lime-hemp plaster (Ty Mawr Lime Ltd., Brecon, UK). All plasterwork was completed by a qualified plasterer who works exclusively in lime plasters.

Simulation of the test cell with the software DesignBuilder™ (Stroud, UK) showed a 1 kW heater would be sufficient to maintain an internal operational temperature of 21°C. This is provided during the heating season November–March by an oil-filled electric radiator thermostatically controlled via an InkBird^®^ (Shenzhen, China) ITC-306 temperature controller, with a set point of 21°C. When heating is in operation, humidification is also provided by a PurLine-Hydro 60™ (Climacity, Madrid, Spain) rotating drum cold water evaporation humidifier, controlled by an InkBird^®^ IHC-200 humidity controller, with a set point of 60% RH. A pedestal-mounted rotating fan is located behind both the heater and humidifier to circulate the air and avoid stratification. Outside of the heating season (i.e., April–October) the internal climatic conditions are free running, with no temperature or humidity control. This replicates the most common conditions within domestic buildings in the UK.

### Interstitial hygrothermal monitoring

The interstitial hygrothermal conditions between materials were monitored at a total of 60 positions. These being, at three depths (interface of internal plaster and insulation, mid-depth, and interface of external plaster and insulation) at the centre of each panel, in the horizontal wall plate at the base of each panel, and halfway up the vertical stud at the junction with the panels finished in NHL 3.5.

**Figure 6:**
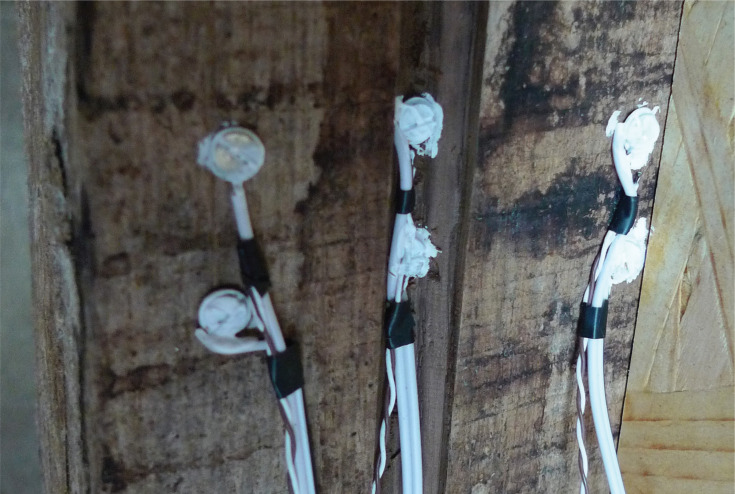
Pairs of stainless-steel screws embedded directly into the timber frame. The electrical resistance between the two is measured and used to calculate the moisture content of the timber.

**Figure 7:**
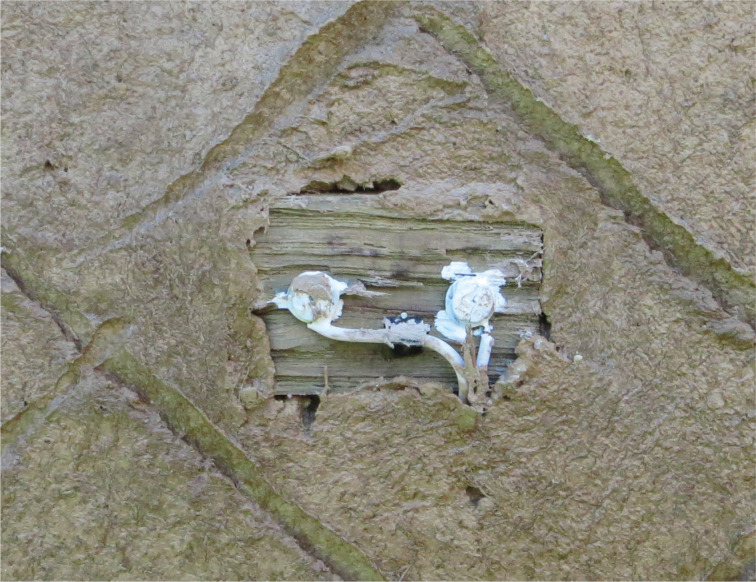
For points at the interface between materials where no timber exists, the stainless-steel screws were embedded in lengths of split oak lath.

Type T thermocouples were used to measure temperature (°C). Following a literature review [4, 20–23], electrical resistance was chosen as the measurement methodology for moisture content (%). This allowed continual measurement with minimal impact on the surrounding materials. A methodology based on the wood block/dowel methodology used by Dr Paul Baker at New Bolsover [3] and reported in Historic England Research Report 43-2016 [24] was followed. Pairs of stainless-steel screws were embedded at the monitoring points, set 20 mm apart along the grain. For monitoring points occurring at the junction between the infill panel and the timber frame, these were embedded directly into the oak frame (Fig. 6). For monitoring points within the depth of the panel, they were embedded in lengths of split oak lath (Fig. 7).

Insulated copper wire connects these back to a Campbell^®^ (Campbell Scientific, Loughborough, UK) CR1000 data logger via a AM16/32 multiplexer. Care was taken in the routing of the wires through the panels to avoid the creation of direct heat and moisture paths. The resistance between the two stainless steel screws is measured by comparing a voltage applied across the screws with that applied across a known resistance (100 kΩ resistor). A calibration exercise was undertaken comparing the resistance measured with gravimetric moisture content measurements of oak blocks at various states between saturated and oven dry. This gave the following equations (1 and 2) for the calculation of the moisture content:

(1) $$\text{If}\;R<0.31225\;\text{Then}\;MC={\left(0.1912R\right)}^{-0.192}$$

(2) $$\text{If}\;R>=0.31225\;\text{Then}\;MC={\left(0.2263R\right)}^{-0.0271}$$

where:

*R* = resistance

*MC* = moisture content %

The measurements must also be corrected for the effect of temperature using equation 3 [25]:

(3) $$MC_{K}=\frac{\left(MC+0.567-0.0260x+0.000051x^{2}\right)}{0.881{\left(1.0056\right)}^{x}}$$

Where:

*MC* = moisture content as measured %

*MC_K_* = temperature corrected moisture content %

*x* = surface temperature +2.8°C

Measurements were recorded at 30-minute intervals. The internal temperature (°C) and RH (%) of the test cell are measured using a Campbell CS215. External temperature (°C), RH (%), precipitation (mm), air pressure (mbar), wind speed (m/s) and wind direction are measured using a Vaisala (Vantaa, Finland) Weather Transmitter WXT520 Series mounted on the roof of the test cell. Direct solar radiation (W/m^2^) incident on the test panels is measured using a Kipp and Zohnen (Delft, Netherlands) CM5 pyrometer.

### Thermal performance

To assess the thermal performance of the replacement infill panels both thermography and in situ U-value measurements were undertaken during the heating seasons of 2019/20 and 2020/21. Thermography was undertaken using a FLIR^®^ (Teledyne FLIR, Oregon, USA) B250 thermal imaging camera. This took place just before dawn, maximising the internal/external temperature difference and avoiding the influence of direct solar gain, on 19/02/20 and 19/11/20. In situ U-value measurements utilised Hukseflux (Delft, Netherlands) HFP01 heat flux plates and type-T thermocouples also connected to a Campbell Scientific CR1000 data logger with readings taken at 5-minute intervals.

## Results

The results presented in this article cover the initial 6-month period 12/12/2019–12/06/2020, during which time the built-in moisture content was drying, and one further year of monitoring post-drying.

### Moisture content

**Figure 8:**
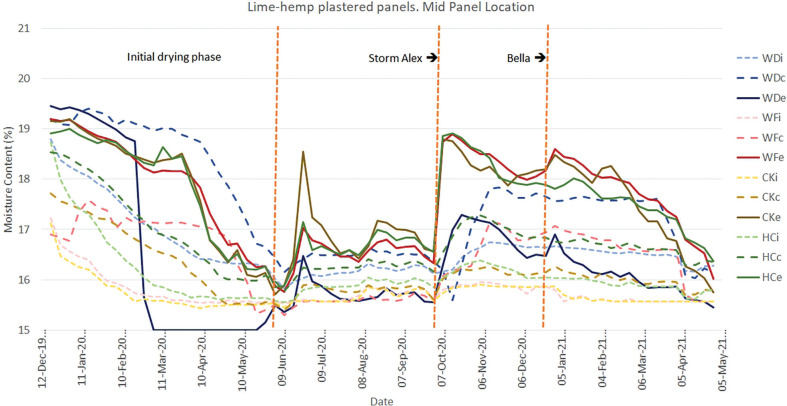
Results for monitoring period 12/12/2019–05/05/2021 for the mid-panel monitoring position for panels finished with lime-hemp plaster. Abbreviations: WD, wattle and daub; WF, wood fibre; CK, cork; HC, hempcrete; i, internal; c, centre; e, external.

**Figure 9:**
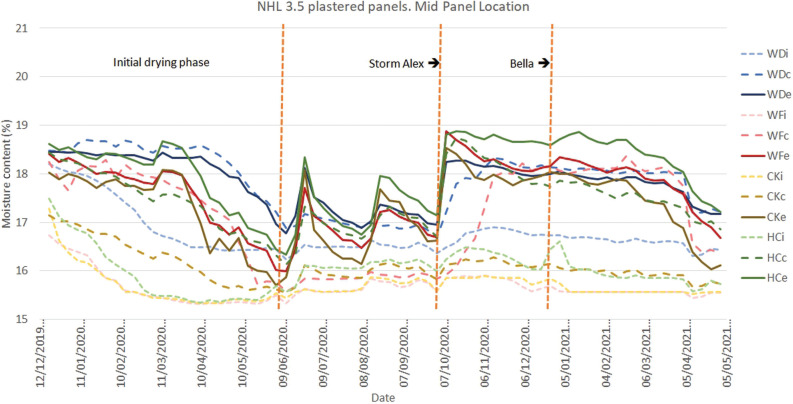
Results for monitoring period 12/12/2019–05/05/2021 for the mid-panel monitoring position for panels finished with NHL 3.5 plaster. Abbreviations: WD, wattle and daub; WF, wood fibre; CK, cork; HC, hempcrete; i, internal; c, centre; e, external.

**Figure 10:**
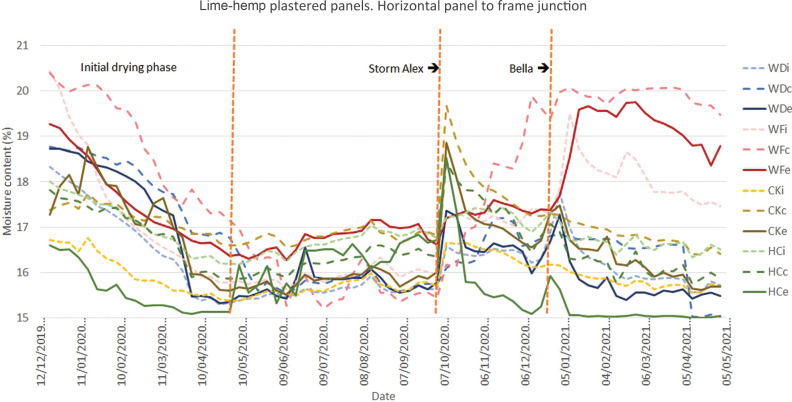
Results for monitoring period 12/12/2019–05/05/2021 for the monitoring position at the horizontal junction between panel and oak frame for panels finished with lime-hemp plaster. Abbreviations: WD, wattle and daub; WF, wood fibre; CK, cork; HC, hempcrete; i, internal; c, centre; e, external.

**Figure 11:**
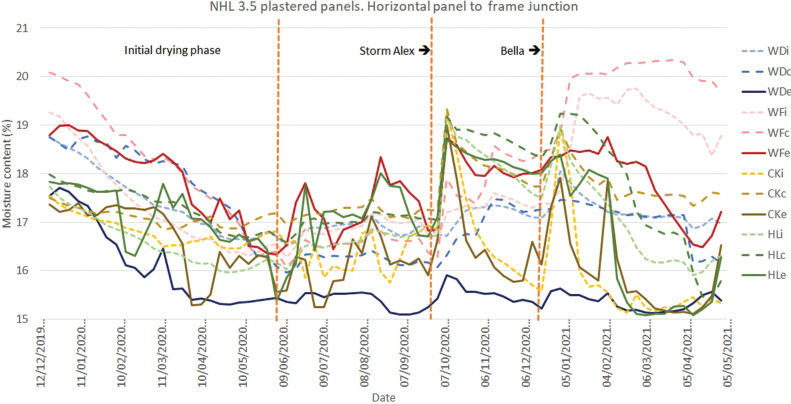
Results for monitoring period 12/12/2019–05/05/2021 for the monitoring position at the horizontal junction between panel and oak frame for panels finished with NHL 3.5 plaster. Abbreviations: WD, wattle and daub; WF, wood fibre; CK, cork; HC, hempcrete; i, internal; c, centre; e, external.

**Figure 12:**
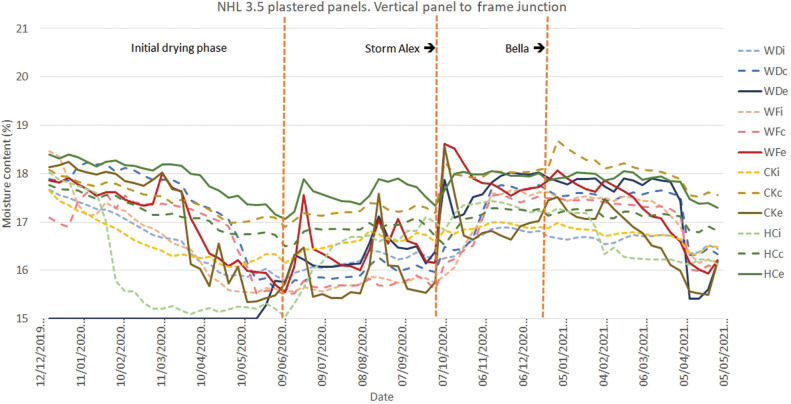
Results for monitoring period 12/12/2019–05/05/2021 for the monitoring position at the vertical junction between panel and oak frame for panels finished with NHL 3.5 plaster. Abbreviations: WD, wattle and daub; WF, wood fibre; CK, cork; HC, hempcrete; i, internal; c, centre; e, external.

The initial results (Figs 8–12) show an initial drying period followed by a series of wetting and drying cycles. To date, no evidence of interstitial condensation has been found, with these wetting cycles correlating with climatic measurements of wind-driven rain. The impact of two major storm events, Alex and Bella, are particularly prominent. Storm Alex was the event that saw a record-breaking wettest day on record [26]. A rapid increase in the moisture content at the interface between the external render and the infill material can be seen in all materials, with the lowest being that of wattle and daub (WDe), for those panels finished in hemp-lime (Fig. 8). It would appear that the lower moisture permeability of the other infill materials concentrates the moisture in the external render, whereas the more moisture permeable wattle and daub allows the moisture to penetrate deeper into the panel. A rise in moisture content being measured at the centre of the panel’s depth (WDc) and at the interface between the infill and internal plaster (WDi) can be seen to follow. Over time, the moisture content at the central position (WDc) becomes higher than the external position (WDe), with this being maintained until the following spring. A similar pattern of behaviour can be seen for the mid-panel positions of the panels finished in NHL 3.5 (Fig. 9).

The results for the monitoring positions located at the junction between the base of the infill panels and the reclaimed oak frame (Figs 10 and 11), show a reduced initial drying period for those panels finished in lime-hemp plaster. Following the storm-induced wetting events, a substantial increase in moisture content is recorded at the centre of the wood wool/wood fibre panel’s depth (WFc). This is followed by an increase at both the internal (WFi) then the external positions (WFe), which continues for the following months. At the centre the moisture content remains around 20%, or more in the case of the panel finished in NHL 3.5, until the spring. The perimeter detail of this panel includes a bitumen impregnated expanding strip and mastic sealant [14], which potentially traps the moisture at this point. This highlights an area for further research and underlines the challenge that this exposed junction presents for both design and workmanship.

The moisture contents at the vertical junction between panel and frame (Fig. 12) are overall lower than those for the horizontal junction (Figs 10 and 11), most probably due to gravity permitting drainage down the joint. The highest moisture content is measured at the centre of the expanded cork board’s depth. An expanding foam sealant is also used here and similarly may be trapping moisture. Further investigation is needed to compare the use of moisture permeable and non-moisture permeable solutions to this interface.

Monitoring is ongoing and it is hoped for a longer period with no major storm events that will allow analysis of the continued drying of the different infill materials.

### Thermal performance

**Figure 13:**
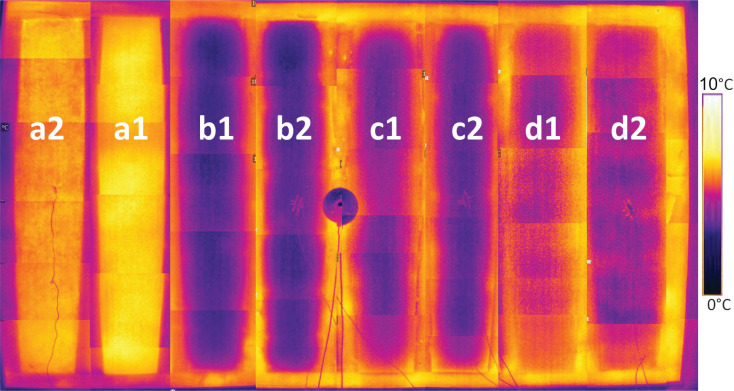
External thermography of test panels. Left to right, a2) wattle and daub with lime-hemp plaster, a1) wattle and daub with NHL 3.5, b1) cork with NHL 3.5, b2) cork with lime-hemp, c1) wood fibre with NHL 3.5, c2) wood fibre with lime-hemp, d1) hempcrete with NHL 3.5 and d2) hempcrete with lime-hemp plaster.

The thermography undertaken on 19/02/20 took place between 6:40 am and 7:20 am. The conditions were as presented in Table 2. The results are compiled as a composite image, as presented in Fig. 13.

**Table 2.** Conditions as measured at the start and finish of thermography 19/02/20

	Time	Ext. temp (°C)	Int. temp (°C)	Δ Temp (°C)	Wind direction (°)	Wind speed (m/s)
Start	06:40	3.63	20.61	16.98	155.6	0.34
Finish	07:20	3.78	20.57	16.79	211.7	0.27

This demonstrates that the panels with the highest external surface temperature, and as such, the poorest thermal performance, are the wattle and daub, with the best performing being the expanded cork board. A small difference in external surface temperature can be seen within the pairs of panels, with those rendered in lime-hemp performing better than those rendered in NHL 3.5. The second round of thermography took place on 19/11/2020 and showed very similar findings, with no significant differences between the two rounds.

The results of the two rounds of in situ U-value measurements (Table 3) corroborated the results obtained by thermography. It had been hoped for a greater difference in moisture content between the first measurements undertaken during the initial drying period and those at the end of the year. However, as noted above, the wetting brought by the storm events resulted in very similar moisture contents. Notwithstanding, there can be seen to be an improvement in the thermal performance of the hempcrete panels over time.

**Table 3.** Results of in situ U-value monitoring showing thermal transmittance of test panels for the periods January–March 2020 and November 2020–January 2021. Best thermal performance highlighted in green, worst in red

Infill material	Internal and external finish	Position	Measured Jan/March 2020 (W/m^2^K)	Measured Nov 2020/Jan 2021 (W/m^2^K)	Change	Calculated (W/m^2^K)	Av. moisture content Jan/March 2020 (%)	Av. moisture content Nov 2020/Jan 2021 (%)	Difference in moisture content (%)
Wattle and Daub	NHL 3.5	Midpoint	2.92	2.95	0.03	2.65	18.2	17.6	−0.6
		Corner	2.18	2.08	−0.10		17.7	16.7	−0.9
	Lime-hemp	Midpoint	2.21	2.39	**0.18**	1.92	18.6	16.9	−1.8
		Corner	2.40	2.38	−0.02		18.0	16.3	−1.7
Cork	NHL 3.5	Midpoint	0.54	0.50	−0.04	0.45	16.8	16.6	−0.2
		Corner	0.68	0.79	0.11		17.2	17.1	−0.1
	Lime-hemp	Midpoint	0.46	0.47	0.01	0.43	17.2	16.6	−0.6
		Corner	0.53	0.53	0.00		17.2	16.5	−0.7
Wood Fibre	NHL 3.5	Midpoint	0.71	0.63	−0.08	0.58	17.3	17.3	0.0
		Corner	0.71	0.79	**0.08**		18.4	18.3	−0.2
	Lime-hemp	Midpoint	0.66	0.66	0.00	0.53	17.3	17.0	−0.4
		Corner	0.77	0.83	0.06		18.4	19.3	1.0
Hempcrete	NHL 3.5	Midpoint	1.56	0.94	−0.62	0.67	17.5	17.6	0.1
		Corner	1.54	1.30	−0.24		17.3	18.3	1.0
	Lime-hemp	Midpoint	1.22	1.00	−0.22	0.58	17.7	16.9	−0.8
		Corner	1.34	1.20	−0.14		16.8	16.1	−0.7

### Biological risks

Work has begun on comparing the measured hygrothermal conditions at each monitoring position with those favourable to the potential biological risks outlined in Table 1. Of the insects, the only significant risk so far identified is from the deathwatch beetle, most frequently towards the inner face of the panels. However, to date no conditions favourable to fungi have been found. As deathwatch beetles require timber to have previously been modified by fungi, the overall risk is lessened. A very small risk from house longhorn beetle and trace risks from powder post beetle and furniture beetle.

## Conclusion

These initial results indicate the relative impact of the moisture permeability of both infill materials and finishing plasters. Specific findings are that no evidence of interstitial condensation in any of the panel build-ups was identified, with increases in moisture content correlating directly with climatic measurements of wind-driven rain. Infill materials with low moisture permeability were seen to produce higher moisture content at the interface with the external render due to the concentration of moisture at this point. Those panels finished in the more moisture permeable lime-hemp plaster, overall present lower moisture contents, with reduced drying times. The use of perimeter, non-moisture permeable, sealants would appear to potentially trap moisture at the junction between infill and historic timber frame. Further research is required into the design and installation of this challenging exposed junction.

The measurements are ongoing and will continue for at least another year. It is hoped that the outcome of this research will assist in the formulation of best practice guidance for the retrofit of historic timber-framed buildings in the UK.
